# Supplementary material for: Pravastatin Prevents Increases in Activity of Metalloproteinase-2 and Oxidative Stress, and Enhances Endothelium-Derived Nitric Oxide-Dependent Vasodilation in Gestational Hypertension
Source: Antioxidants (Basel). 2023 Apr 16;12(4):939. doi: 10.3390/antiox12040939 (PMC10135677; doi:10.3390/antiox12040939)
Supplement: Supplementary file 1 [file antioxidants-12-00939-s001.zip › antioxidants-2316258-supplementary.pdf]

Supplementary Table S1. Average of maternal, fetal placental and biochemical parameters.

Parameter	Norm-Preg	Norm-Preg+Prava	HTN-Preg	HTN-Preg+Prava
Systolic blood pressure (mmHg)	110 ± 2	109 ± 1	129 ± 2*	102 ± 1
Litter size (Number of pups)	9 ± 1	9 ± 2	11 ± 2	10 ± 1
Placental weight (g)	0.28 ± 0.01	0.29 ± 0.01	0.22 ± 0.01*	0.28 ± 0.01
Plasma levels of NO metabolites (mmol/L)	0.30 ± 0.03	0.31 ± 0.02	0.22 ± 0.01*	0.29 ± 0.02
Lipid peroxide levels (mmol/L)	0.11 ± 0.007	0.12 ± 0.005	0.17 ± 0.012*	0.13 ± 0.003
Antioxidant capacity (mmol Trolox Equiv./L)	0.17 ± 0.011	0.19 ± 0.019	0.07 ± 0.011*	0.19 ± 0.17
Activity of 75 KDa MMP-2 (arbitrary units)	0.049 ± 0.008	0.050 ± 0.006	0.046 ± 0.007	0.045 ± 0.007
Activity of 72 KDa MMP-2 (arbitrary units)	0.295 ± 0.025	0.0237 ± 0.019	0.247 ± 0.011	0.253 ± 0.019
Activity of 65 KDa MMP-2 (arbitrary units)	0.073 ± 0.011	0.073 ± 0.006	0.100 ± 0.009*	0.068 ± 0.006
ACh R_{max} (%)	80.2 ± 5.4	85.1 ± 4.1	77.9 ± 6.8	85.5 ± 7.2
ACh pEC_{50} (-log M)	6.80 ± 0.12	7.69 ± 0.17#	6.83 ± 0.30	7.97 ± 0.14#

Data are expressed as means ± SEM in Norm-Preg (n = 8), Norm-Preg+Prava (n = 8), HTN-Preg (n = 8) and HTN-Preg+Prava (n= 8) groups. *P < 0.05 vs Norm-Preg group, and #P < 0.05 vs. Norm-Preg and HTN-Preg groups.

Zymography gel 1 of placenta

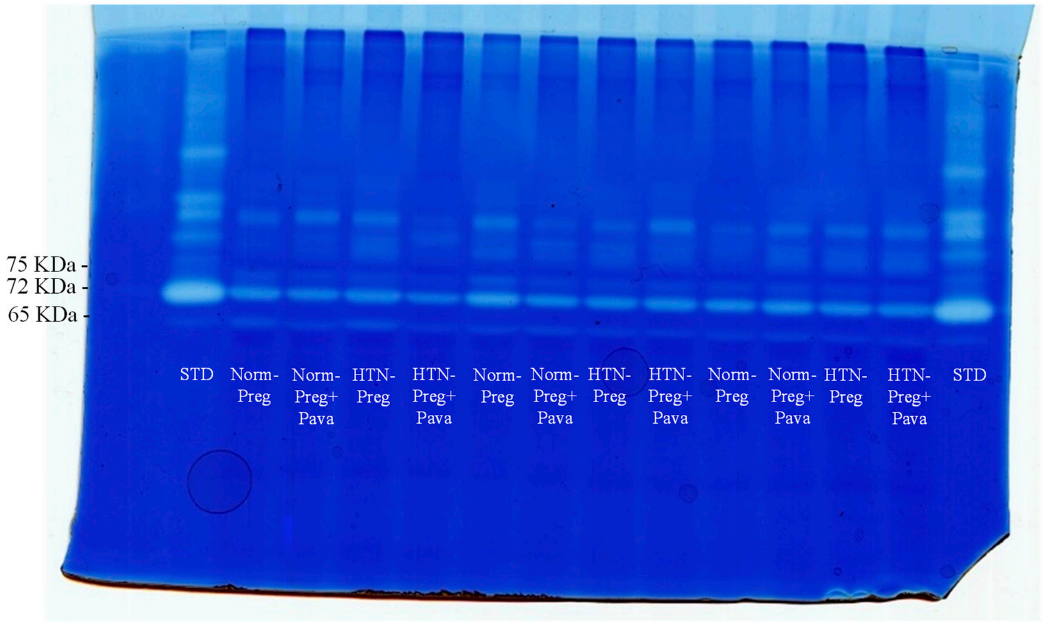

Zymography gel 2 of placenta

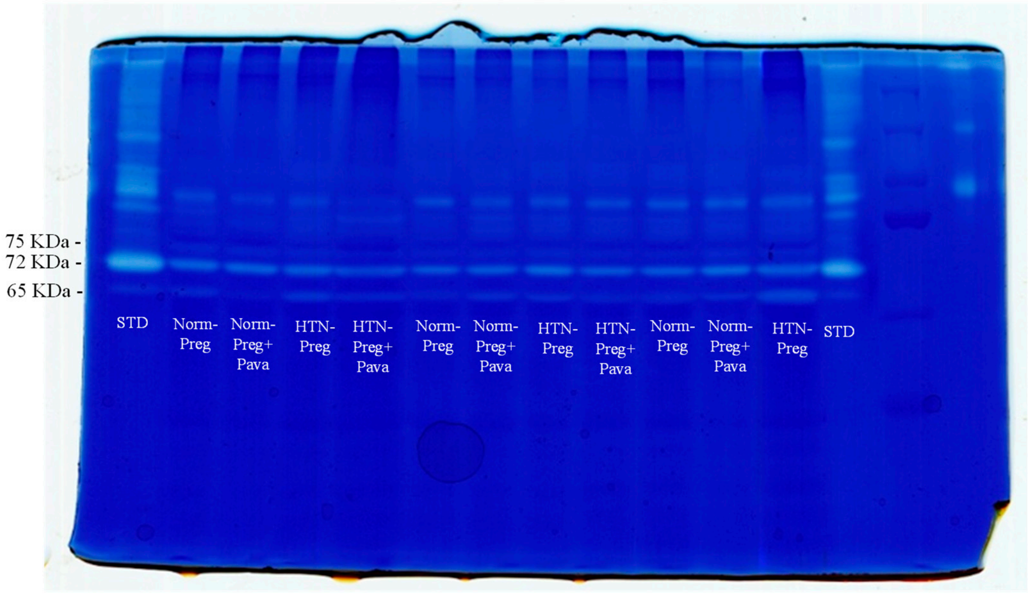

Supplementary Figure S1. Acrylamide gels (1 and 2) stained with Coomassie Brilliant Blue to quantify gelatinolytic activities of 75 KDa MMP-2, 72 KDa MMP-2 and 65 KDa MMP-2 in placenta samples from Norm-Preg, Norm-Preg+Prava, HTN-Preg and HTN-Preg+Prava groups. STD: internal standard.
